# The number of key carcinogenic events can be predicted from cancer incidence

**DOI:** 10.1038/s41598-017-12448-7

**Published:** 2017-09-22

**Authors:** Aleksey V. Belikov

**Affiliations:** School of Biological and Medical Physics, Laboratory of Innovative Medicine and Agrobiotechnology, Moscow Institute of Physics and Technology (MIPT), Institutsky per., 9, 141701 Dolgoprudny, Moscow Region, Russia

## Abstract

The widely accepted multiple-hit hypothesis of carcinogenesis states that cancers arise after several successive events. However, no consensus has been reached on the quantity and nature of these events, although “driver” mutations or epimutations are considered the most probable candidates. By using the largest publicly available cancer incidence statistics (20 million cases), I show that incidence of 20 most prevalent cancer types in relation to patients’ age closely follows the Erlang probability distribution (R^2^ = 0.9734–0.9999). The Erlang distribution describes the probability *y* of *k* independent random events occurring by the time *x*, but not earlier or later, with events happening on average every *b* time intervals. This fits well with the multiple-hit hypothesis and potentially allows to predict the number *k* of key carcinogenic events and the average time interval *b* between them, for each cancer type. Moreover, the amplitude parameter *A* likely predicts the maximal populational susceptibility to a given type of cancer. These parameters are estimated for 20 most common cancer types and provide numerical reference points for experimental research on cancer development.

## Introduction

The value of cancer incidence and mortality curves for inferring information about the underlying carcinogenic processes has long been recognized^1^. It has been the basis for the influential multiple-hit hypothesis of cancer development, which proposed that cancer appears after several consecutive mutations^2–4^. That prediction was based on the assumption that cancer mortality increases proportionally to the n-th power of age. However, already at that time it was known that many cancers display deceleration of mortality growth at an advanced age, which could not be explained by the power law. Many complicated equations based on multiple assumptions and empirically estimated parameters have since been proposed, attempting to model the limited growth of cancerous cells^5–7^. However, current data unequivocally show that cancer incidence not only ceases to increase with age but, for at least some cancers, decreases^8,9^. This behaviour cannot be explained by growth equations and has been puzzling biologists and clinicians for considerable time. The depletion of susceptible population, decreased exposure to carcinogens and conversion of cells to the proliferation-arrested, senescent phenotype have been put forward as some of the possible explanations, but none have been convincingly demonstrated (see Discussion in ref.^9^).

Here I propose that cancer incidence by age is, in fact, a statistical distribution of probabilities that a required number of carcinogenic events occurs by the given age, but not earlier or later. Of 16 tested continuous distributions, the best fit is observed for the gamma distribution and its special case – the Erlang distribution. Notably, these two distributions describe the probability of several independent random events occurring precisely by the given time. This takes the multiple-hit hypothesis to a new level and allows to estimate the number of key carcinogenic events and the average time interval between them, for each cancer type. Moreover, the amplitude parameter of generalized probability distributions likely predicts the maximal populational susceptibility to a given type of cancer. The Erlang distribution exhibits the excellent fit to incidence of each of 20 most prevalent cancer types, with the average R^2^ of 0.995. The estimated parameters suggest high heterogeneity in the carcinogenesis process and populational susceptibility amongst cancer types and provide reference points for experimental research.

## Results

The probability density function (PDF) is used to specify the probability of a random variable falling within a particular range of values. This probability is given by the integral of this variable’s PDF over that range. In the context of cancer incidence, such integral would specify the probability of a patient’s age at a cancer diagnosis falling within a particular age group. It can be seen that such PDF integrates to 1, because once a cancer is diagnosed, the patient must belong to one and only to one of the age groups. For the purpose of elucidating the underlying nature of cancer, this *incidence* PDF should be calculated with the assumption of indefinite 100% survival of the population, to exclude the confounding influence of *mortality* from a given cancer type, other cancer types, other diseases and other causes, and thus can be called mortality-independent incidence. It is important to note that it does not specify the probability to be *diagnosed* with cancer at a particular age (for those who survived to that age), because such function would not integrate to 1 (not every person is to develop cancer during his lifetime, especially a particular type of cancer). However, the latter probability can be derived from the former after multiplying by the maximal populational susceptibility to a given type of cancer. This parameter estimates what fraction of the population would develop a given type of cancer if the population would live indefinitely (reflecting age- and mortality-independent incidence). For an individual person, this parameter describes the maximal probability to develop a given type of cancer during his lifetime or, in other words, the probability of having a susceptible genotype and living in a permissive environment. Thus, the probability to be diagnosed with a particular cancer type at a particular age (for those who survived to that age) is the product of the maximal probability to be diagnosed with this cancer type at all during lifetime and the mortality-independent probability of the age at the cancer diagnosis falling within this age group.

How can the probability to be diagnosed with cancer at a particular age (for those who survived to that age) be calculated from empirical data? It is logical to suggest that the number of newly diagnosed (during the year of observation) cancer cases in a particular age group normalized by the total number of living people in that age group would reflect this probability. In epidemiological terms, it is the crude incidence rate of a particular age group. Such accounting for the number of survivors until a particular age is very important to focus on true incidence of a given type of cancer without confounding influence of mortality from various causes. Because each person is counted only once in each age group (at his actual age during the year of observation), there is no need to normalize by the length of age intervals to derive PDF (an incidence rate for the “50 to 60 y.o.” age group would be approximately equal to an incidence rate for the single “55 y.o.” age). Likewise, when the data are pooled from several consecutive years of observation, each person that is counted several times in the denominator also has proportionally more years to develop cancer, so no adjustment is needed as well.

**Figure 1:**
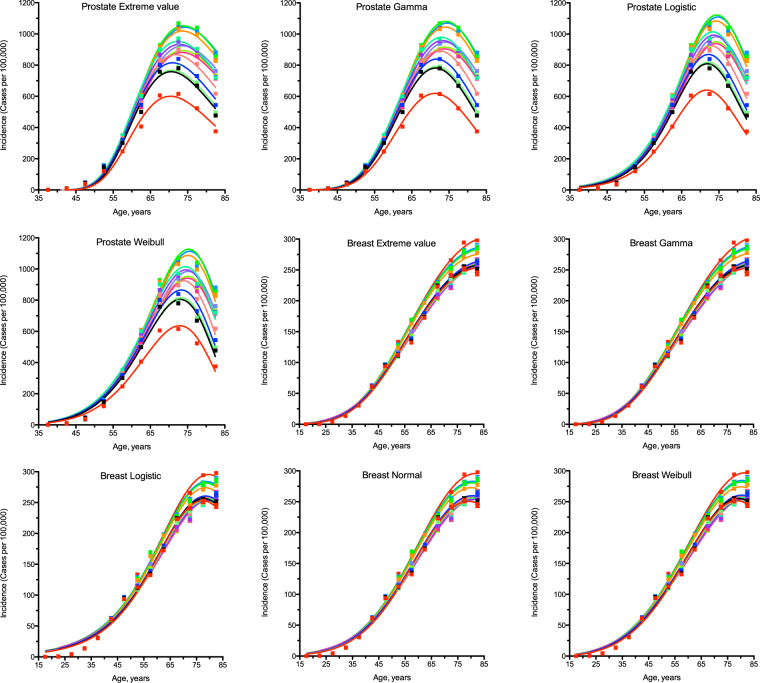
Comparison of different statistical distributions with actual distributions of prostate and breast cancer incidence by age. Dots indicate actual data for 5-year age intervals, curves indicate PDFs fitted to the data. The middle age of each age group is plotted. Different colours indicate different years of observation, from 1999 to 2012. The fitting procedure was identical for all distributions. The normal distribution did not converge for prostate cancer. Prostate and breast cancers were selected due to being the highest-incidence gender-specific cancer types.

To test the probability hypothesis, the latest publicly available USA cancer incidence data were downloaded from the CDC WONDER database (see Methods for details and Supplementary Data 1–50 for original downloads). The PDFs for the general forms of the following continuous probability distributions were tested for fit with least squares non-weighted nonlinear regression analysis: beta, Cauchy, extreme value, Fisher F, gamma, Gompertz, chi-square, Levy, logistic, Maxwell, normal, Rayleigh, Student t, Wald and Weibull (see Methods for details and Supplementary Data 21 for the original project file). Only the extreme value, gamma, logistic, normal and Weibull distributions provided acceptable fits for most of cancer types. Whilst the gamma distribution has only a marginal advantage in the goodness of fit amongst five selected distributions when incidence for different genders and years of observation is combined (see Supplementary Table S1 for R^2^ and Supplementary Table S2 for standard deviation of the residuals), it shows systematically superior fits when gender-specific cancers are evaluated separately for each observation year (Fig. 1, see Supplementary Tables S3 and S4 for R^2^, Supplementary Fig. S1 for the residual plots, Supplementary Tables S5 and S6 for the standard deviation of the residuals, Supplementary Tables S7 and S8 for the Akaike Information Criterion, and Supplementary Data 50 for the original project file). Interestingly, the gamma distribution has been used before to estimate confidence intervals for age-adjusted cancer rates^10–12^.

Most importantly, the gamma distribution and the Erlang distribution derived from it are the only classical continuous probability distributions that describe the cumulative waiting time for *k* successive random events, with the Erlang distribution differing only in counting events as integer numbers. Because these properties suit excellently to describe the waiting time for real discrete random events such as mutations, the Erlang distribution provides the opportunity to get unique insights into the carcinogenesis process. I propose that the shape parameter *k* of the Erlang distribution indicates the average number of key carcinogenic events that need to occur in order for a cancer to develop to a stage that can be detected during clinical screening. The scale parameter *b* indicates the average time interval (in years) between such events. Finally, the amplitude parameter *A* divided by 1000 estimates the maximal susceptibility (in percent) of a given population to a given type of cancer. This is because the area under the PDF curve is always unity, the maximal area under the cancer incidence curve is 100,000 (cases per 100,000 people), and *A* is used to convert probability into incidence.

**Figure 2:**
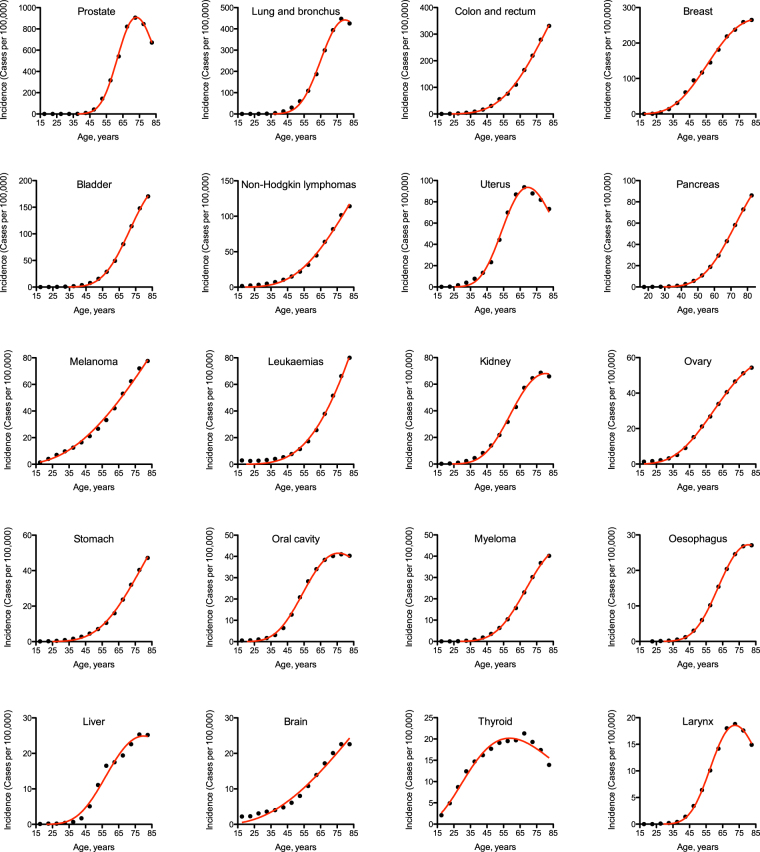
The Erlang distribution approximates cancer incidence by age for 20 most prevalent cancer types. Dots indicate actual data for 5-year age intervals, curves indicate the PDF of the Erlang distribution fitted to the data (see Table 1 for R^2^ and estimated parameters). The middle age of each age group is plotted. Cancer types are arranged in the order of decreasing incidence.

To obtain these parameter values, the Erlang distribution was fitted individually to incidence of each of 20 most prevalent cancer types (Fig. 2, Table 1, see Methods for details and Supplementary Data 21 for the original project file). The goodness of fit varied from 0.9734, for thyroid cancer, to 0.9999, for pancreatic and oesophageal cancers, with the average of 0.9953. The predicted number of carcinogenic events varied from 4, for melanoma and brain cancer, to 41, for prostate cancer. The predicted average time between the events varied from 2 years, for prostate cancer, to 81 years, for melanoma. The predicted maximal populational susceptibility varied from 1%, for oesophageal, hepatic and laryngeal cancers, to 100%, for melanoma. Overall, the data predict high heterogeneity in carcinogenesis patterns.

**Table 1:** Estimated carcinogenesis parameters for 20 most prevalent cancer types.

Cancer type	*k*	*b*	*A/1000*	R^2^
	Number of carcinogenic events ± s.e.m.	Average time between events, years ± s.e.m.	Maximal populational susceptibility, % ± s.e.m.	Goodness of fit
Prostate	41 ± 1	1.83 ± 0.00	26.40 ± 0.18	0.9992
Lung and bronchus	30 ± 2	2.75 ± 0.01	16.44 ± 0.24	0.9981
Colon and rectum	10 ± 1	13.75 ± 0.17	66.93 ± 3.80	0.9991
Breast	9 ± 1	10.71 ± 0.09	20.44 ± 0.46	0.9981
Bladder	21 ± 1	4.59 ± 0.02	9.93 ± 0.17	0.9995
Non-Hodgkin lymphomas	8 ± 1	19.26 ± 0.58	31.21 ± 3.90	0.9964
Uterus	20 ± 1	3.67 ± 0.02	3.77 ± 0.05	0.9954
Pancreas	15 ± 1	7.07 ± 0.01	7.15 ± 0.06	0.9999
Melanoma	4 ± 1	81.01 ± 7.38	100	0.9954
Leukaemias	8 ± 2	23.56 ± 1.09	49.57 ± 10.93	0.9957
Kidney	15 ± 1	5.75 ± 0.04	3.69 ± 0.07	0.9971
Ovary	8 ± 1	13.66 ± 0.12	5.40 ± 0.13	0.9989
Stomach	11 ± 1	11.51 ± 0.15	7.25 ± 0.42	0.9986
Oral cavity	13 ± 1	6.32 ± 0.03	2.29 ± 0.03	0.9983
Myeloma	16 ± 1	6.14 ± 0.03	2.67 ± 0.06	0.9992
Oesophagus	20 ± 0	4.25 ± 0.00	1.27 ± 0.00	0.9999
Liver	13 ± 2	6.67 ± 0.11	1.45 ± 0.07	0.9863
Brain	4 ± 1	76.69 ± 13.77	26.34 ± 14.52	0.9777
Thyroid	5 ± 0	14.67 ± 0.24	1.52 ± 0.04	0.9734
Larynx	24 ± 1	3.15 ± 0.01	0.71 ± 0.01	0.9989

The parameters are determined for the Erlang distribution fitted to actual cancer incidence data (see Fig. 2). Cancer types are listed in the order of decreasing incidence.

To evaluate reproducibility and robustness of parameter estimation, the gamma distribution was fitted to incidence of prostate cancer separately for each observation year (Fig. 1, Table 2, see Supplementary Data 50 for the original project file). The gamma distribution was selected instead of the Erlang distribution to show precise estimates for the number of carcinogenic events. Prostate cancer was selected due to the highest incidence, the highly efficient screening procedure, the highest estimated number of carcinogenic events and the dramatic variation in incidence between the years of observation. Whilst the maximal populational susceptibility dropped from 32% in 1999 to 17% in 2012 (by 47%), which is explained largely by the official 2008 and 2011/2012 recommendations against screening^13–15^, the average time between events varied from 1.6 to 2.0 years (±11%) and the estimated number of carcinogenic events varied from 38 to 45 (±8%). The upward trend in the number of carcinogenic events may be readily explained by the detection of cancer at later stages in the absence of early screening^13–15^. The variation before the 2008 recommendation was only from 38 to 41 events (±3.8%). Such robustness in the estimation of the number of carcinogenic events for a given cancer type highlights its fundamental nature and thus lends further support to the multiple-hit hypothesis of carcinogenesis.

**Table 2:** Robustness of carcinogenesis parameter estimation for prostate cancer.

Year of observation	*k*	*b*	*A*/*1000*	R^2^
	Number of carcinogenic events ± s.e.m.	Average time between events, years ± s.e.m.	Maximal populational susceptibility, % ± s.e.m.	Goodness of fit
1999	40.72 ± 1.28	1.876 ± 0.063	31.79 ± 0.48	0.9992
2000	39.56 ± 1.28	1.931 ± 0.067	32.23 ± 0.50	0.9992
2001	40.59 ± 1.16	1.873 ± 0.057	32.00 ± 0.43	0.9993
2002	38.82 ± 0.99	1.955 ± 0.053	31.57 ± 0.38	0.9994
2003	38.37 ± 1.25	1.981 ± 0.069	28.82 ± 0.45	0.9991
2004	38.10 ± 1.41	1.992 ± 0.079	27.94 ± 0.49	0.9988
2005	38.67 ± 1.29	1.959 ± 0.070	27.33 ± 0.43	0.9990
2006	39.85 ± 1.21	1.886 ± 0.061	28.30 ± 0.39	0.9991
2007	40.14 ± 1.46	1.863 ± 0.072	28.67 ± 0.47	0.9987
2008	41.56 ± 1.58	1.784 ± 0.072	25.49 ± 0.43	0.9984
2009	42.91 ± 1.79	1.711 ± 0.075	23.35 ± 0.42	0.9979
2010	44.39 ± 2.16	1.651 ± 0.084	21.62 ± 0.45	0.9971
2011	44.97 ± 2.48	1.623 ± 0.094	21.14 ± 0. 50	0.9962
2012	44.19 ± 2.32	1.648 ± 0.090	16.84 ± 0.38	0.9964

The parameters are determined for the gamma distribution fitted to actual cancer incidence data (see Fig. 1). The gamma distribution was selected instead of the Erlang distribution to show precise estimates for the number of carcinogenic events. Prostate cancer was selected due to the highest incidence, the highly efficient screening procedure, the highest estimated number of carcinogenic events and the dramatic variation in incidence between the years of observation.

## Discussion

I have shown that cancer incidence by age is best approximated by the Erlang distribution. In most general sense, the Erlang distribution is the sum of *k* independent exponentially distributed random variables, each of which has the mean of *b*. As in the case of cancer incidence by age the argument *x* corresponds to time, and the exponential distribution describes the time between events in a Poisson process, i.e. a process in which events occur independently at a constant average rate, the Erlang distribution describes the cumulative waiting time for *k* successive random events. Therefore, the only assumption that is required for a mechanistic interpretation is that key carcinogenic events occur independently at a constant average rate. This assumption does not appear unreasonable, as mutations in individual genes are indeed random events that occur independently of each other usually at a constant average rate defined by the levels of gamma and UV radiation, reactive oxygen species and carcinogenic substances, and the rates of DNA replication and repair. While these parameters may vary during the adult lifespan, they do not appear to substantially change in a monotonic and unidirectional fashion, and instead fluctuate around an average.

The DNA replication rate may increase during uncontrolled tumour growth. However, it happens at the last stage (terminal clonal expansion) after most, if not all, key mutations have occurred^16^. The key mutations may accumulate silently. Indeed, accumulation of mutations and realization of their potential are two different processes that may occur relatively independently. Mutations can stay dormant for a long time, as exemplified by latent driver mutations that exert their effects only upon the occurrence of another mutation or other favourable condition^17^. Another possibility is that a mutation can occur in a gene responsible for DNA replication, DNA repair or antioxidant activity, thus increasing the overall mutation rate. However, mutations in such “mutator” genes are found in only about 15–20% of tumour samples^18,19^, and thus are not an essential feature of carcinogenesis^20^ and cannot have a major influence on cancer incidence statistics. In fact, given the excellent fit of the Erlang distribution to the actual data, this assumption of mutation randomness can be viewed as the prediction, i.e. that previous mutations do not substantially affect the appearance rate of subsequent mutations.

The progression from one carcinogenesis stage to the other is usually assumed to be mediated by “driver” mutations in crucial genes, which give the mutated cell some growth advantage, apoptosis resistance or other oncogenic properties, as opposed to inconsequential “passenger” mutations^21^. Many algorithms have been suggested for identification of driver mutations^22^, indicating that no universally accepted criteria exist. Moreover, whilst hundreds of potential driver mutations have been identified in various tumours, they need not be all present in the same tumour specimen, as many of them are redundant or even mutually exclusive, e.g. when the affected proteins are components of the same signalling pathway^23^. Thus, each tumour is expected to have only a sample of all possible driver mutations. Another aspect to consider is that while one mutation is usually sufficient to activate an oncogene, two mutations are typically required to inactivate both alleles of a tumour suppressor gene. Therefore, the number of carcinogenetic events predicted by the Erlang distribution should be translated not into the number of mutated driver genes, but rather into the number of driver mutations.

When cancer drivers are searched for in tumour genomes, most studies focus on nonsynonymous point mutations^24^. This gives relatively low numbers of driver mutations, in the range from one to eight (Fig. 3 in ref.^24^). However, it has been recently shown that synonymous^25^ and noncoding^26^ mutations also can act as carcinogenesis drivers. Moreover, there are many more types of genetic alterations that can possibly contribute to cancer progression. They include indels^27^, homozygous deletions^28^, inversions^29^, tandem duplications^30^, amplifications^31^, intra- and inter-chromosomal translocations^32^ (often resulting in gene fusions^33^), as well as chromosomal arm-level and whole-level copy-number alterations^34^, and chromothripsis^35^. Additionally, epigenetic alterations (epimutations) are a whole new level of potential cancer drivers^36,37^.

It is likely that many of these alterations contribute to progression of each cancer type. Moreover, different cancer types and subtypes require different proportions of these alterations^38^, e.g. some cancers are driven mostly by point mutations, some by amplifications, yet some by gene fusions. Interestingly, the total number of important alterations per tumour ranged from 0 to 40 (Fig. 2c in ref.^38^), which corresponds to the range of event numbers predicted by the Erlang distribution. Therefore, the number of carcinogenic events per tumour predicted by the current theory is most likely the sum of driver alterations of several different types. Astonishingly, the recent massive omics study of 333 primary prostate carcinomas by The Cancer Genome Atlas Research Network has found only a single or no alterations in up to 26% of tumour samples^19^. In extreme case, this may mean that the true nature of carcinogenesis drivers is still not known.

Most data that were used in this study represent combined cancer cases, e.g. acute and chronic, lymphocytic, myeloid and monocytic leukaemias were combined into Leukaemias. The resulting curve is necessary different in shape, position and amplitude from the curves of individual leukaemia subtypes. Hence, the estimated parameters are also different and reflect only the average. When the exact number of carcinogenic alterations is required, it is necessary to analyse the data for a particular cancer subtype and also for a particular gender and race. Such data are readily accessible at the CDC WONDER portal.

Another factor that influences the results is the stage at which cancer is diagnosed. Cancer types that are diagnosed at early stages, e.g. due to highly developed screening programs, will likely undergo fewer carcinogenic transformations by the time of the first diagnosis than cancers that are difficult to diagnose early. Thus, the current theory predicts the average number of carcinogenic events that happen by the time of diagnosis and not by the time of appearance of the first malignant cell or the time of full cancer development. Therefore, improvements in diagnostics will likely lead to decreases in the estimated numbers of carcinogenic events. A curious counterexample with abandonment of efficient screening that led to an increase in the number of carcinogenic events has been provided in the Results section.

Overall, the theory and methodology presented here allow to generate testable predictions about the carcinogenesis process in any cancer subtype for which reliable incidence statistics is available. Thus, they may help to define the subtype-specific cancer drivers, by providing numerical reference points. Also, the estimated maximal populational susceptibility may help to predict the allele frequencies of driver genes. Finally, these findings provide additional support to the multiple-hit theory of carcinogenesis.

## Methods

### Data acquisition

United States Cancer Statistics Public Information Data: Incidence 1999–2012 were downloaded via Centers for Disease Control and Prevention Wide-ranging OnLine Data for Epidemiologic Research (CDC WONDER) online database (http://wonder.cdc.gov/cancer-v2012.HTML). The United States Cancer Statistics (USCS) are the official federal statistics on cancer incidence from registries having high-quality data for 50 states and the District of Columbia. Data are provided by The Centers for Disease Control and Prevention National Program of Cancer Registries (NPCR) and The National Cancer Institute Surveillance, Epidemiology and End Results (SEER) program. Results were grouped by 5-year Age Groups, Crude Rates were selected as output, and all other settings were kept at default values. Crude Rates are expressed as the number of cases reported each calendar year per 100,000 population. A single person with more than one primary cancer verified by a medical doctor is counted as a case report for each type of primary cancer reported. The population estimates for the denominators of incidence rates are a slight modification of the annual time series of July 1 county population estimates (by age, sex, race, and Hispanic origin) aggregated to the state or metropolitan area level and produced by the Population Estimates Program of the U.S. Bureau of the Census (Census Bureau) with support from the National Cancer Institute (NCI) through an interagency agreement. These estimates are considered to reflect the average population of a defined geographic area for a calendar year. The data were downloaded separately for each cancer type, upon its selection in the Leading Cancer Sites tab. The original txt downloads are available as Supplementary Data 1–20. For the parameter estimation robustness test, the data for prostate and breast cancers were additionally downloaded separately for each year of observation. These txt downloads are available as Supplementary Data 22–49.

### Data selection and analysis

For analysis, the data were imported into GraphPad Prism 5. The following age groups were selected: “15–19 years”, “20–24 years”, “25–29 years”, “30–34 years”, “35–39 years”, “40–44 years”, “45–49 years”, “50–54 years”, “55–59 years”, “60–64 years “, “65–69 years”, “70–74 years”, “75–79 years” and “80–84 years”. Prior age groups were excluded due to unreliably low incidence rates, and “85+ years” was excluded due to the undefined age interval. The middle age of each age group was used as the x value, e.g. 17.5 for the “15–19 years” age group. Data were analysed with Nonlinear regression. The following User-defined equations were created for the statistical distributions:

*Extreme value:*

Y = A*(exp(−((x−t)/b)))*(exp(−exp(−((x−t)/b))))/b

*Gamma:*

Y = A*(x^(k−1))*(exp(−x/b))/((b^k)*gamma(k))

*Logistic:*

Y = A*(exp((x−t)/b))/(b*((1 + exp((x−t)/b))^2))

*Normal:*

Y = A*(exp(−0.5*(((x−t)/b)^2)))/(b*((2*pi)^0.5))

*Weibull:*

Y = A*(k/(b^k))*(x^(k−1))*exp(−((x/b)^k))

The parameter *A* was constrained to “Must be between zero and 100000.0”, parameter *t* to “Must be between zero and 150.0”, parameters *b* and *k* to “Must be greater than 0.0”. “Initial values, to be fit” for all parameters were set to 1.0. All other settings were kept at default values, e.g. Least squares fit and No weighting.

For the Erlang distribution, the parameter *k* for each cancer type was estimated by the fitting of the Gamma distribution, rounded to the nearest integer and used as “Constant equal to” in the second round of the Gamma distribution fitting, which provided the final results.

The original pzfx project file with data, analyses and graphs is available as Supplementary Data 21. The pzfx project file for the parameter estimation robustness test is available as Supplementary Data 50.

## Electronic supplementary material

Supplementary Information

Supplementary Data 1-50
