# Supplementary material for: The number of key carcinogenic events can be predicted from cancer incidence
Source: Sci Rep. 2017 Sep 22;7:12170. doi: 10.1038/s41598-017-12448-7 (PMC5610194; doi:10.1038/s41598-017-12448-7)
Supplement: Supplementary file 1 — Supplementary Information [file 41598_2017_12448_MOESM1_ESM.pdf]

The number of key carcinogenic events can be predicted from cancer incidence
Aleksey V. Belikov

Supplementary Table S1. Comparison of the goodness of fit (R^2) of different statistical distributions to actual distributions of cancer incidence by age.

Cancer type	Extreme value	Gamma	Logistic	Normal	Weibull
Prostate	0.9960	**0.9992**	0.9954	0.9990	0.9922
Lung and bronchus	0.9964	0.9981	0.9994	0.9994	**0.9995**
Colon and rectum	0.9992	0.9991	0.9992	0.9995	**0.9996**
Breast	0.9980	**0.9982**	0.9905	0.9961	0.9965
Bladder	0.9993	0.9995	0.9994	**0.9999**	**0.9999**
Non-Hodgkin lymphomas	0.9971	0.9965	**0.9997**	0.9982	0.9981
Uterus	**0.9957**	0.9954	0.9849	0.9918	0.9830
Pancreas	**0.9999**	**0.9999**	0.9986	**0.9999**	0.9997
Melanoma	0.9975	0.9963	**0.9987**	0.9981	0.9970
Leukaemias	0.9964	0.9957	**0.9986**	0.9971	0.9966
Kidney	0.9963	0.9972	0.9988	0.9993	**0.9995**
Ovary	**0.9991**	0.9989	0.9959	0.9988	0.9987
Stomach	0.9988	0.9986	**0.9999**	0.9995	0.9996
Oral cavity	**0.9990**	0.9984	0.9867	0.9948	0.9915
Myeloma	0.9991	0.9992	0.9993	NC	**0.9999**
Oesophagus	0.9997	**0.9999**	0.9960	0.9996	0.9978
Liver	**0.9873**	0.9863	0.9700	0.9803	0.9774
Brain	0.9828	0.9779	**0.9924**	0.9861	0.9808
Thyroid	0.9709	**0.9745**	0.9452	0.9608	0.9726
Larynx	0.9962	**0.9989**	0.9959	NC	0.9957
**Average**	0.9952	**0.9954**	0.9922	0.9943	0.9938

Cancer types are listed in the order of decreasing incidence. The best fit for each cancer type is highlighted in bold. NC – not converged.

The number of key carcinogenic events can be predicted from cancer incidence
Aleksey V. Belikov

Supplementary Table S2. Comparison of the standard deviation of the residuals (Sy.x) of different statistical distributions fitted to actual distributions of cancer incidence by age.

Cancer type	Extreme value	Gamma	Logistic	Normal	Weibull
Prostate	25.14	**11.62**	27.08	12.90	35.39
Lung and bronchus	11.51	8.325	4.555	4.602	**4.078**
Colon and rectum	3.380	3.635	3.482	2.698	**2.501**
Breast	4.980	**4.624**	10.72	6.900	6.530
Bladder	1.707	1.483	1.553	**0.6948**	0.7928
Non-Hodgkin lymphomas	2.281	2.531	**0.6876**	1.782	1.857
Uterus	**2.716**	2.828	5.114	3.772	5.422
Pancreas	0.2555	0.2557	1.197	**0.2553**	0.5343
Melanoma	1.424	1.709	**1.002**	1.231	1.538
Leukaemias	1.713	1.871	**1.056**	1.530	1.651
Kidney	1.794	1.577	1.035	0.7812	**0.6379**
Ovary	**0.6286**	0.7051	1.369	0.7335	0.7754
Stomach	0.6028	0.6505	**0.2149**	0.3979	0.3404
Oral cavity	**0.5949**	0.7436	2.151	1.351	1.722
Myeloma	0.4878	0.4480	0.4295	NC	**0.1174**
Oesophagus	0.2079	**0.0905**	0.7637	0.2535	0.5634
Liver	**1.256**	1.304	1.934	1.567	1.678
Brain	1.104	1.251	**0.7343**	0.9922	1.166
Thyroid	1.087	**1.019**	1.492	1.262	1.056
Larynx	0.5138	**0.2734**	0.5373	NC	0.5493
**Average**	3.169	**2.347**	3.355	2.428	3.445

Cancer types are listed in the order of decreasing incidence. The best fit for each cancer type is highlighted in bold. NC – not converged.

The number of key carcinogenic events can be predicted from cancer incidence
Aleksey V. Belikov

Supplementary Table S3. Comparison of the goodness of fit (R^2) of different statistical distributions to actual distributions of prostate cancer incidence by age.

Year of observation	Extreme value	Gamma	Logistic	Normal	Weibull
1999	0.9974	**0.9992**	0.9920	NC	0.9885
2000	0.9972	**0.9992**	0.9921	NC	0.9886
2001	0.9960	**0.9993**	0.9939	NC	0.9907
2002	0.9972	**0.9994**	0.9921	NC	0.9886
2003	0.9971	**0.9991**	0.9914	NC	0.9876
2004	0.9967	**0.9988**	0.9911	NC	0.9872
2005	0.9964	**0.9990**	0.9921	NC	0.9882
2006	0.9959	**0.9991**	0.9923	NC	0.9874
2007	0.9949	**0.9987**	0.9922	NC	0.9868
2008	0.9924	**0.9984**	0.9940	NC	0.9880
2009	0.9906	**0.9979**	0.9939	NC	0.9859
2010	0.9881	**0.9971**	0.9948	NC	0.9872
2011	0.9862	**0.9962**	0.9946	NC	0.9860
2012	0.9869	**0.9964**	0.9941	NC	0.9849
**Average**	0.9936	**0.9984**	0.9929	-	0.9875

The best fit for each year of observation is highlighted in bold. NC – not converged.

The number of key carcinogenic events can be predicted from cancer incidence
Aleksey V. Belikov

Supplementary Table S4. Comparison of the goodness of fit (R^2) of different statistical distributions to actual distributions of breast cancer incidence by age.

Year of observation	Extreme value	Gamma	Logistic	Normal	Weibull
1999	0.9978	**0.9982**	0.9888	0.9951	0.9955
2000	0.9985	**0.9988**	0.9878	0.9951	0.9952
2001	0.9987	**0.9989**	0.9887	0.9957	0.9957
2002	0.9990	**0.9992**	0.9888	0.9959	0.9959
2003	0.9976	**0.9981**	0.9869	0.9942	0.9948
2004	0.9974	**0.9981**	0.9869	0.9940	0.9949
2005	0.9967	**0.9974**	0.9864	0.9934	0.9942
2006	0.9971	**0.9976**	0.9882	0.9945	0.9951
2007	0.9967	**0.9970**	0.9882	0.9943	0.9947
2008	0.9963	**0.9967**	0.9888	0.9944	0.9948
2009	0.9961	**0.9963**	0.9899	0.9948	0.9952
2010	0.9952	0.9954	0.9917	0.9952	**0.9957**
2011	0.9925	0.9927	0.9905	NC	**0.9940**
2012	0.9925	0.9926	0.9910	0.9935	**0.9943**
**Average**	0.9966	**0.9968**	0.9888	0.9946	0.9950

The best fit for each year of observation is highlighted in bold. NC – not converged.

The number of key carcinogenic events can be predicted from cancer incidence

Aleksey V. Belikov

Supplementary Figure S1. Residual plots for the Figure 1: Comparison of different statistical distributions with actual distributions of prostate and breast cancer incidence by age.

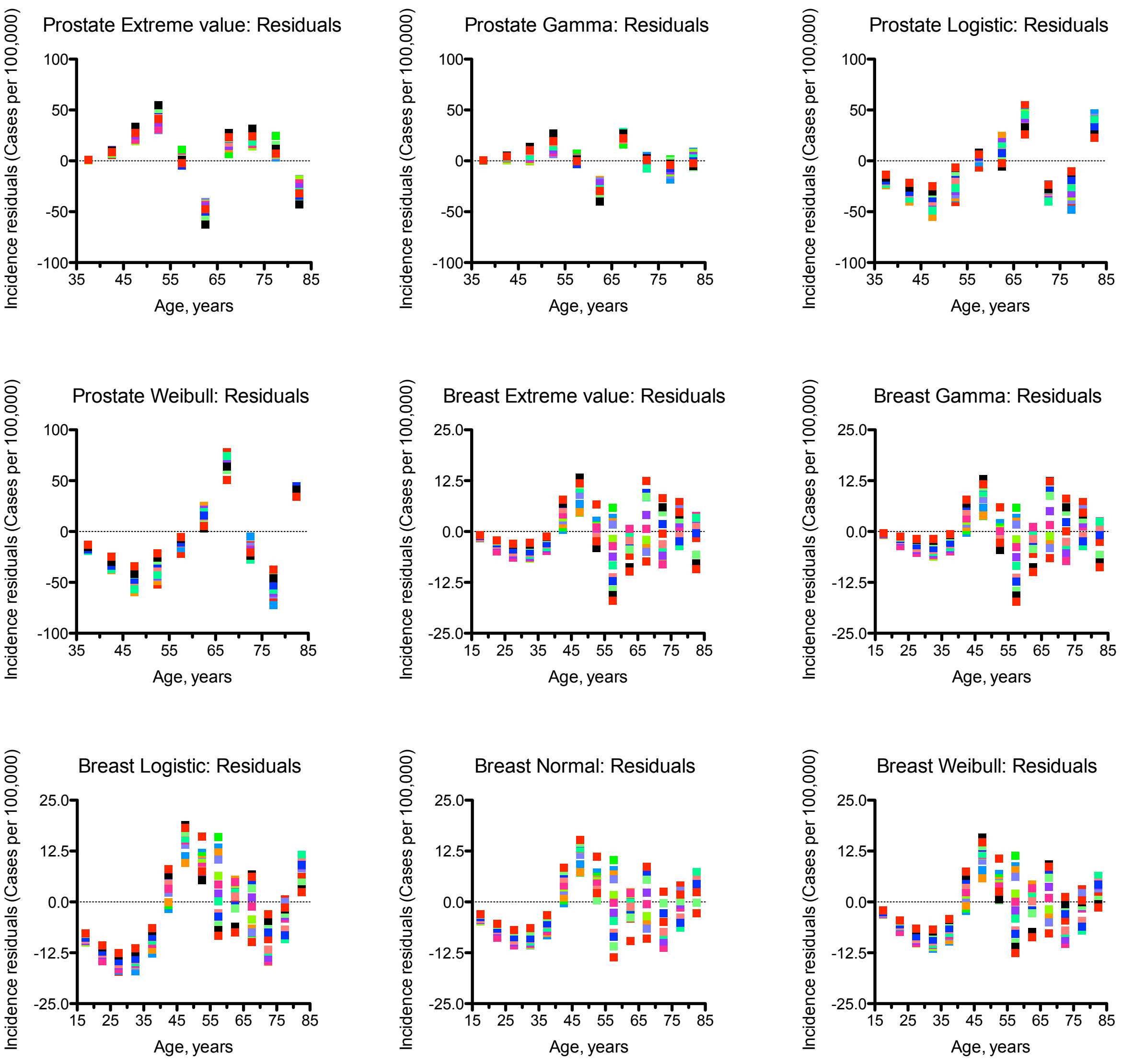

Dots indicate residuals (the difference between the actual data for 5-year age intervals and the values obtained from PDFs fitted to the data). The middle age of each age group is plotted. Different colours indicate different years of observation, from 1999 to 2012. The fitting procedure was identical for all distributions. The normal distribution did not converge for prostate cancer. Prostate and breast cancers were selected due to being the highest-incidence gender-specific cancer types.

The number of key carcinogenic events can be predicted from cancer incidence
Aleksey V. Belikov

Supplementary Table S5. Comparison of the standard deviation of the residuals (Sy.x) of different statistical distributions fitted to actual distributions of prostate cancer incidence by age.

Year of observation	Extreme value	Gamma	Logistic	Normal	Weibull
1999	25.73	**14.24**	45.09	NC	54.12
2000	26.61	**14.62**	44.60	NC	53.72
2001	32.02	**13.37**	39.61	NC	48.72
2002	25.93	**11.54**	43.43	NC	52.12
2003	23.68	**13.37**	40.95	NC	49.27
2004	24.49	**14.77**	40.29	NC	48.32
2005	25.37	**13.28**	37.43	NC	45.70
2006	28.36	**13.17**	38.84	NC	49.77
2007	32.32	**16.42**	39.92	NC	51.74
2008	35.52	**16.25**	31.55	NC	44.66
2009	36.75	**17.31**	29.51	NC	44.98
2010	38.82	**19.19**	25.71	NC	40.38
2011	41.16	**21.74**	25.87	NC	41.45
2012	31.64	**16.50**	21.30	NC	34.01
**Average**	30.60	**15.41**	36.01	-	47.07

The best fit for each year of observation is highlighted in bold. NC – not converged.

The number of key carcinogenic events can be predicted from cancer incidence
Aleksey V. Belikov

Supplementary Table S6. Comparison of the standard deviation of the residuals (Sy.x) of different statistical distributions fitted to actual distributions of breast cancer incidence by age.

Year of observation	Extreme value	Gamma	Logistic	Normal	Weibull
1999	5.742	**5.194**	12.98	8.554	8.200
2000	4.565	**4.034**	13.12	8.296	8.226
2001	4.337	**3.891**	12.62	7.833	7.747
2002	3.710	**3.292**	12.24	7.420	7.449
2003	5.263	**4.645**	12.22	8.159	7.695
2004	5.353	**4.638**	12.06	8.132	7.547
2005	6.013	**5.306**	12.26	8.560	7.979
2006	5.675	**5.212**	11.50	7.847	7.396
2007	6.158	**5.849**	11.69	8.104	7.839
2008	6.545	**6.268**	11.49	8.127	7.802
2009	6.838	**6.647**	11.02	7.910	7.617
2010	7.350	7.244	9.672	7.327	**6.935**
2011	9.258	9.158	10.47	NC	**8.336**
2012	9.275	9.170	10.14	8.594	**8.080**
**Average**	6.149	**5.753**	11.68	8.066	7.775

The best fit for each year of observation is highlighted in bold. NC – not converged.

The number of key carcinogenic events can be predicted from cancer incidence
Aleksey V. Belikov

Supplementary Table S7. The probabilities that different statistical distributions fit better than gamma distribution to actual distributions of prostate cancer incidence by age, calculated according to the Akaike Information Criterion (AIC).

Year of observation	Extreme value	Logistic	Normal	Weibull
1999	0.27%	<0.01%	NC	<0.01%
2000	0.25%	<0.01%	NC	<0.01%
2001	0.02%	<0.01%	NC	<0.01%
2002	0.03%	<0.01%	NC	<0.01%
2003	0.33%	<0.01%	NC	<0.01%
2004	0.63%	<0.01%	NC	<0.01%
2005	0.15%	<0.01%	NC	<0.01%
2006	0.05%	<0.01%	NC	<0.01%
2007	0.11%	<0.01%	NC	<0.01%
2008	0.04%	0.13%	NC	<0.01%
2009	0.05%	0.48%	NC	<0.01%
2010	0.09%	5.09%	NC	0.06%
2011	0.17%	14.97%	NC	0.16%
2012	0.15%	7.24%	NC	0.07%
**Average**	0.18%	2.00%	-	0.03%

NC – not converged.

The number of key carcinogenic events can be predicted from cancer incidence
Aleksey V. Belikov

Supplementary Table S8. The probabilities that different statistical distributions fit better than gamma distribution to actual distributions of breast cancer incidence by age, calculated according to the Akaike Information Criterion (AIC).

Year of observation	Extreme value	Logistic	Normal	Weibull
1999	21.35%	<0.01%	0.15%	0.26%
2000	16.68%	<0.01%	<0.01%	<0.01%
2001	17.96%	<0.01%	<0.01%	<0.01%
2002	15.81%	<0.01%	<0.01%	<0.01%
2003	14.80%	<0.01%	0.04%	0.09%
2004	11.84%	<0.01%	0.04%	0.11%
2005	16.42%	<0.01%	0.20%	0.50%
2006	23.31%	<0.01%	0.32%	0.74%
2007	32.75%	<0.01%	1.03%	1.63%
2008	35.28%	0.02%	2.57%	4.45%
2009	40.19%	0.08%	8.05%	12.92%
2010	44.93%	1.72%	45.98%	**64.77%**
2011	45.45%	14.89%	NC	**77.25%**
2012	46.03%	19.64%	**71.27%**	**85.48%**
**Average**	27.34%	2.60%	9.98%	17.73%

When an alternative model is preferred, the probability is highlighted in bold. NC – not converged.
